# Space-valence mapping of social concepts: Do we arrange negative and positive ethnic stereotypes from left to right?

**DOI:** 10.3389/fpsyg.2022.1070177

**Published:** 2022-12-09

**Authors:** K. Kühne, K. Nenaschew, A. Miklashevsky

**Affiliations:** ^1^Potsdam Embodied Cognition Group, Cognitive Sciences, University of Potsdam, Potsdam, Germany; ^2^Faculty of Psychology, FernUniversität in Hagen, Hagen, Germany

**Keywords:** body-specificity hypothesis, embodied cognition, ethnic stereotypes, in-group stereotypes, implicit associations, GNAT, out-group stereotypes

## Abstract

**Introduction:**

The body-specificity hypothesis states that in right-handers, positive concepts should be associated with the right side and negative concepts with the left side of the body. Following this hypothesis, our study postulated that negative out-group ethnic stereotypes would be associated with the left side, and positive in-group stereotypes would be associated with the right side.

**Methods:**

The experiment consisted of two parts. First, we measured the spatial mapping of ethnic stereotypes by using a sensibility judgment task, in which participants had to decide whether a sentence was sensible or not by pressing either a left or a right key. The sentences included German vs. Arabic proper names. Second, we measured implicit ethnic stereotypes in the same participants using the Go/No-go Association Task (GNAT), in which Arabic vs. German proper names were presented in combination with positive vs. negative adjectives. Right-handed German native speakers (N = 92) participated in an online study.

**Results:**

As predicted, in the GNAT, participants reacted faster to German names combined with positive adjectives and to Arabic names combined with negative adjectives, which is diagnostic of existing valenced in-and outgroup ethnic stereotypes. However, we failed to find any reliable effects in the sensibility judgment task, i.e., there was no evidence of spatial mapping of positive and negative ethnic stereotypes. There was no correlation between the results of the two tasks at the individual level. Further Bayesian analysis and exploratory analysis in the left-handed subsample (N = 9) corroborated the evidence in favor of null results.

**Discussion:**

Our study suggests that ethnic stereotypes are not automatically mapped in a body-specific manner.

## Introduction

Embodied cognition theory claims that the body is involved in conceptual processing. For example, understanding perception-and action-related language, such as words *red*, *loud*, *pick*, or *kick*, leads to activation of sensorimotor representations (e.g., Pecher et al., 2004; Fischer and Zwaan, 2008; see also Hauk et al., 2004; and Bernabeu et al., 2017, for neuroscientific evidence). However, it remains particularly challenging for the embodied framework to explain processing of abstract concepts (Borghi, 2020), such as valence, as well as the role of embodied representations in complex social cognition. In this study, we examined the existence of automatic embodied mapping of positive and negative social stereotypes on the horizontal axis (i.e., from left to right), as predicted by the body-specificity hypothesis (Casasanto, 2011).

### The body-specificity hypothesis

Around a decade ago, Casasanto (2009) found a strong association between valence and handedness. Namely, in right-handers, positive concepts are associated with the right side and negative concepts with the left side of the body. For left-handers, the pattern is the opposite. Linguistic metaphors often align with this finding: The right is associated with positive concepts, while the left is associated with negative concepts. Metaphors like “the right hand of the king” or “having two left feet” can be found in many languages. Similarly, space-valence associations are reflected in cultural behaviors, such as entering the church with the right foot or entering the bathroom with the left foot (Casasanto, 2009). However, this lateralization goes beyond purely cultural traditions. This pervasive space-valence mapping is explained by a *body-specificity hypothesis*. The body-specificity hypothesis claims that individual bodily experiences resulting from unique body configuration (e.g., body size or weight) and functionality (e.g., dominant side or vision and hearing abilities) shape individual cognitive representations (Casasanto, 2011).

In a series of experiments conducted by Casasanto (2009), participants were instructed to place a drawn animal either to the left or right, depending on whether the participants thought the animal was good or bad. Most right-handers drew the “good” animal on the right side and the “bad” animal on the left side. Left-handers demonstrated an opposite pattern. These findings also held when participants responded verbally, i.e., without using their hands. In another experiment, participants evaluated personal characteristics (e.g., intelligence, attractiveness, honesty, or happiness) of fictive alien characters as more positive when those aliens were drawn on the side of the page corresponding to the participant’s dominant hand (Casasanto, 2009). In a similar task, the findings by Casasanto were replicated with footedness (Weber and Sun, 2020). Strikingly, five-year-old children already demonstrate these implicit associations between space and valence (Casasanto and Henetz, 2012). Moreover, space-valence associations were also found in the vertical and saggital dimensions (Marmolejo-Ramos et al., 2013, 2018).

The initial findings by Casasanto (2009) inspired a new line of research investigating space-valence associations in various domains and at different cognitive levels. Space-valence associations were found in visual recognition: A 100-euro banknote (having a more positive connotation) was better recognized in the right visual field than a 5-euro banknote (Giuliani et al., 2018). The coding of affective information is also influenced by handedness: Whereas right-handers remembered more stimuli located on the right side, the opposite was true for left-handers (Brunyé et al., 2012). Interestingly, space-valence associations also seem to hold for the auditory domain: Right-handers tended to experience more positive emotions when music was presented to their right ear, as McFarland and Kennison (1989) found.

The relationship between space and valence is bidirectional: Not only lateralized perception and action influence cognition, but also valenced cognitive representations might influence perception and action. In a study by Fernández et al. (2019), positive biographic memories facilitated more rightward movements, and negative biographic memories facilitated more leftward movements. Similarly, participants look longer to the right while listening to positive information and to the left when listening to negative information (Çatak et al., 2018).

Importantly, space-valence associations influence explicit judgments. Right-handed participants judged facial expressions as more negative when those expressions were presented in the left visual field; the participants also judged facial mood as more positive when it was presented in the right visual field (Natale et al., 1983). At the same time, left-handers evaluated neutral stimuli as more positive when those stimuli were presented in the left visual field (Everhart et al., 1996). In another experiment, participants had to evaluate the performance of skiers in videos from a dual mogul competition where two skiers simultaneously moved downhill side by side (Loffing et al., 2019). Again, the participants favored the skiers on their dominant side. Right-handers also prefer buying products and hiring job applicants presented on their right side (Casasanto, 2009).

Finally, space-valence associations have also been demonstrated in multimodal communicative processes in real-life settings. Analyzing videos from the final debates of the 2004 and 2008 U.S. presidential elections, Casasanto and Jasmin (2010) found that positive speech was associated with right-hand gestures in the two right-handed candidates (Bush and Kerry), while the negative speech was associated with left-hand gestures. The opposite pattern was found in the two left-handed candidates (McCain and Obama).

Only a few studies examined space-valence associations with linguistic stimuli. In four experiments, de la Vega et al. (2012) studied whether positive concepts are associated with the dominant hand and negative concepts with the non-dominant hand. In Experiment 1, right-handers were asked to make a lexical decision by pressing one key with their right hand for words and another key with their left hand for pseudowords. The authors found faster right-hand responses to all stimuli, regardless of valence. In Experiment 2, participants made a valence judgment, i.e., explicitly responded to positive vs. negative words with the right or left key. This time, the authors found a reliable interaction between emotional valence and response side. In Experiment 3, the task was the same as in Experiment 2 but with left-handed participants. The pattern of results was the opposite of Experiment 2: Left-handers responded faster to positive words with their left hand and to negative words with their right hand. Finally, in Experiment 4, participants were asked to perform a valence judgment but in a Go/No-go paradigm, i.e., to respond only to positive or to negative stimuli. Again, no interaction between valence and response side emerged. The authors conclude that valence-space horizontal associations do not appear automatically but require a task with both an explicit valence judgment and explicit response mapping to occur.

In another study, right-and left-handed participants were asked to discriminate emotional valence of positive and negative words (Kong, 2013, Experiment 1). The author found space-valence compatibility effects similar to the results by de la Vega et al. (2012): Namely, right-handers were faster when responding to positive words with the right hand and to negative words with the left hand. Left-handers demonstrated the opposite pattern, as predicted by the body-specificity hypothesis.

The mechanisms behind space-valence associations still remain unclear. One suggested explanation for the space-valence mapping is brain lateralization of approach-avoidance behaviors (Brookshire and Casasanto, 2012): Approach reactions are associated with activity in the left frontal lobe (controlling the right side of the body), and avoidance reactions are associated with activity in the right frontal lobe (controlling the left side of the body). Accordingly, the “good is right” and “bad is left” conceptual associations might be grounded in lateralized approach-avoidance behaviors with their neural correlates.

Another possible explanation is motor fluency: Participants are more skilled in using their dominant hand and thus associate the dominant hand with more fluency and positive outcomes. Once participants cannot use their dominant hand easily, for example, due to a stroke or even an inconvenient ski glove being worn for a short time, participants’ standard space-valence associations weaken (Casasanto, 2011; see also Fuente et al., 2017).

### Stereotypes measurement

Despite a variety of paradigms and researched domains, little is known about horizontal associations of complex social concepts, such as stereotypes. Stereotypes are mental simplifications of complex characteristics or actions of certain groups of people (Allport, 1954). As such, stereotyping is not a negative phenomenon: It helps humans to orient themselves in complex reality and to automate everyday decisions. Through stereotyping, a sense of belonging to a particular group and thus social identity develops, enhancing self-esteem (Tajfel and Turner, 1986).

With respect to one’s social group, three types of stereotypes can be defined: auto-stereotypes (***in-group***, what we think of our own group), meta-stereotypes (what we think others think of our group), and hetero-stereotypes (***out-group***, what we think of the other group; Marin and Salazar, 1985; Tajfel and Forgas, 2000). The out-group stereotypes are often negative images of others, which might represent an obstacle to intercultural communication. Negative out-group stereotypes of ethnicities, genders, or specific other groups may have extensive consequences for personal decisions and the entire society. For example, job candidates of Turkish origin are less likely to receive a positive call-back in a job application procedure in Germany and the Netherlands (Thijssen et al., 2021; for similar examples from other contexts, see Correll et al., 2007; Fiske and Lee, 2008; Price and Wolfers, 2010; González et al., 2019).

The first effort to measure stereotypes was made in 1933 by simply assigning characteristics from a list of 84 adjectives to different races^1^ (Katz and Braly, 1933). This method is known as an “adjective selection technique,” “checklist technique” or “typicality rating.” It was used in multiple studies and was even proclaimed “exemplar” in stereotype research (Ashmore and Del Boca, 1979). However, the procedure was criticized in several respects as being too simplistic, prone to artifacts, and unable to pinpoint fine-grained differentiations between categories (Hamilton and Trolier, 1986).

An alternative method was the Brigham method (“percentage rating” or “diagnostic ratio”), which reported subjective assumptions about the frequency distribution of certain traits in specific groups of persons (Brigham, 1971; see also McCauley and Stitt, 1978). However, again, mere calculation of the mean frequency values of certain attributes for a group is doomed to ambivalent results. Further methods working with adjectives or characteristics were introduced, such as “semantic differential,” “polarity profile,” or the “Gardner method” (Gardner et al., 1988; Osgood, 2009).

Crucially, most of the above methods used explicit measures to evaluate stereotypes. It can be a problem in this sensitive research area: Participants may be unaware of their true opinion (Banaji and Greenwald, 1994), or they may be reluctant to disclose negative feelings toward a specific group (social desirability effect, Crosby et al., 1980). In an attempt to overcome this drawback of explicit methods, Jones and Sigall (1971) developed a bogus pipeline technique based on elaborate deception of participants: The researchers claimed that the device used in the experiment could identify if participants lie. Obviously, this method is undesirable for ethical reasons.

Finally, different scales were introduced, such as the Modern Racism Scale (McConahay, 1986) or the more recent Perceived Online Racism Scale (Keum and Miller, 2017). In these questionnaires, participants are asked to what extent they agree with covertly racist statements, such as “Over the past few years, Blacks have gotten more economically than they deserved” (McConahay, 1986), or whether participants experienced discrimination in online communication, e.g., “In the past 6 months, I have received a racist meme” (Keum and Miller, 2017). A disadvantage of such surveys is that participants might tend to give socially acceptable answers, especially in the case of normative topics. There is often a discrepancy between the “true” attitudes of the respondents and the views endorsed by social norms (Stocké, 2004). Participants with negative out-group stereotypes may be concerned about potential consequences for themselves, for example, at school or at work, if they demonstrate those negative stereotypes. This tendency can also differ across groups depending on the salience of certain norms in those groups. While xenophobic attitudes are socially considered taboo or at least undesirable in most modern societies, in certain communities, such as right-wing movements, xenophobic attitudes might be conversely praised.

To overcome limitations of explicit measurement methods, implicit methods can be used, such as the Go/No-go Association Task (GNAT), a variation of the Implicit Association Test (IAT, Nosek and Banaji, 2001). It measures the strength of association between a target category (e.g., a social group) and one of the two poles of an attribute dimension (good-bad). This task requires a key press in response to both a target category and a target attribute. In the GNAT paradigm, participants respond to one type of stimuli (Go) but not to another (No-go). For example, in one experimental block, participants can be asked to press the key if they see a Black person or a positive attribute (incongruent condition) but refrain from responding if they see either a White person or a negative attribute. In another experimental block, participants can be asked to press the key if they see either a Black person or a negative attribute (congruent condition) but refrain from responding when they see a White person or a positive attribute. The difference in accuracy and reaction times between these two conditions, i.e., incongruent and congruent, is assumed to reflect the strength of the implicit association between Black people and negative attributes.

Implicit methods should not rely on introspective access to the measured constructs and thus presumably limit conscious control over the response. Previous studies have successfully utilized either IAT (Gawronski, 2002; Ratliff and Smith, 2021) or GNAT (Calanchini et al., 2021) to reveal social stereotypes. The IAT was proved to be a valid instrument for assessing stereotype strength (Gawronski, 2002).

Despite its name, the implicit association test and its variation, GNAT, is not genuinely implicit: Participants are explicitly asked to respond to valenced stimuli (good or bad) in combination with a specific category. This fact might reveal the experiment’s true purpose, and it is still possible that participants exhibit conscious control of their reactions (*cf.* Stefanutti et al., 2020). Indeed, research showed that participants were aware of their implicit responses and attitudes when asked to predict their results on upcoming IAT (Hahn et al., 2014; Hahn and Gawronski, 2019).

Several studies have shown that implicitly recorded attitudes only moderately correlate with explicit responses for the “same” construct (Nosek, 2005; Gschwendner et al., 2006; but see Nosek and Smyth, 2007); and the relationship between implicit associations and real-life behaviors is complex and indirect (Kurdi et al., 2019). Another critique of IAT/GNAT is that it measures shallow linguistic associations rather than deeper conceptual processing (Lynott et al., 2012).

Last but not least, more critique is coming from the translational point of view: Although implicit associations, as measured by the IAT, can be intentionally modified by training (Forscher et al., 2019), this modification does not necessarily translate into a modification of explicit behaviors. Therefore, further methods tapping into implicit associations should be explored, possibly with higher applied potential (e.g., Suitner et al., 2017; *cf.* Brusa et al., 2021).

### Present study

As the body-specificity hypothesis predicts, right-handers should associate positive in-group stereotypes with the right-hand responses and negative out-group stereotypes with the left-hand responses. The goal of the present study was to examine this hypothesis.

The current study employed an implicit and conceptually demanding task as a possible tool to assess the automatic spatial mapping of social stereotypes: a sensibility judgment (SJ) task. Under automatic, we understand here associations that are activated regardless of the task or context, i.e., when neither space nor valence is explicitly mentioned in the task. We used a modification of the SJ task inspired by the body-specificity hypothesis. In our version of the SJ task, participants were asked to judge whether a sentence was semantically plausible (e.g., *Ibrahim steers a boat.*) or not (e.g., *Charlotte eats a hammer.*) by pressing either the left or right response key. We used German vs. Arabic names for agents in sentences to induce in-and out-group stereotypes in German participants. Participants’ attention was not directly drawn to valence or ethnic stereotypes, and thus the true purpose of the experiment remained unraveled. In this case, any observed space-valence associations would be considered automatic, i.e., activated, despite their irrelevance to the task.

We used the GNAT after the SJ task as a well-established tool to assess stereotypes in our participants. In the GNAT, the target category was “foreigners” (represented by Arabic names), and the participants were successively presented with target concept stimuli (German vs. Arabic names) combined with valenced attributes (positive vs. negative adjectives).

Thus, in line with the body-specificity hypothesis, we posit that, in German right-handed participants, positive in-group stereotypes about Germans should be mapped on the right side, and negative out-group stereotypes about Arabs should be mapped on the left side.

## Materials and methods

### Stimuli materials

Thirty German names were selected from the list of Germany’s most popular given names in 2020 (Vornamen 2020: Emilia und Noah auf Platz 1|GfdS, 2021). Thirty names were selected from the list of Germany’s most frequent Arabic and/or Turkish names (Rodríguez, 2010). Thirty sensible [e.g., *Susanne (Samira) isst einen Kuchen.* /Susanne (Samira) is eating a cake.] and 30 non-sensible sentences[e.g., *Nico (Yağmur) trinkt einen Handschuh.* /Nico (Yağmur) is drinking a glove.] were constructed in German. The agent always appeared in the first position in a sentence.

For the GNAT, 30 positive and 30 negative German adjectives were taken from the study by Bluemke and Friese (2006). All stimuli materials can be found in Appendix A (see Supplementary material).

### Design and procedure

The experiments were programmed and run using the online Gorilla Experiment Builder service (Anwyl-Irvine et al., 2020).^2^ All participants started with the SJ task. Participants were presented with a fixation cross (250 ms) followed by a sentence (until response, timed out after 3,000 ms). In half of the trials, meaningful sentences were presented, and meaningless sentences appeared in the other half of trials. German vs. Turkish/Arabic names were used with equal probability for agents in sentences. There was no fixed correspondence between the names and content of sentences. Instead, the experiment software always randomly combined names with the remaining parts of the sentences (see Table 1 for examples).

**Table 1:** List of example trials in the SJ task.

Condition	Example trials	English translation
German & sensible	Karl fährt ein Auto.	Karl drives a car.
German & non-sensible	Karl fährt ein Sofa.	Karl drives a sofa.
German & sensible	Charlotte isst einen Kuchen.	Charlotte eats a cake.
German & non-sensible	Charlotte isst einen Hammer.	Charlotte eats a hammer.
Arabic & sensible	Meryem spricht Spanisch.	Meryem speaks Spanish.
Arabic & non-sensible	Meryem spricht Wasser.	Meryem speaks water.
Arabic & sensible	Ibrahim steuert ein Boot.	Ibrahim steers a boat.
Arabic & non-sensible	Ibrahim steuert ein Vacuum.	Ibrahim steers a vacuum.

Keys Q and P (i.e., located on the left and right, respectively) were used for responses to meaningful vs. meaningless sentences. Reverse mapping was used in another block, and the order of blocks was counterbalanced across participants. Each block started with a practice consisting of 10 trials, followed by 120 experimental trials, i.e., there were 240 experimental trials in the entire SJ task. During practice, participants received feedback: Green ticks appeared in the screen center for correct responses, and red crosses indicated errors. No feedback was provided in experimental trials. After every 30 trials, participants were suggested to take a break. Stimuli were presented in black on a white background; the font Sans Serif (14 pixels) was used. Instructions used in the experiment can be found in online Supplementary material (see Data availability statement).

After completion of the SJ task, participants proceeded with the GNAT. Four blocks in the GNAT corresponded to four possible response rules or go-conditions: (1) Arabic names combined with negative adjectives, (2) Arabic names combined with positive adjectives, (3) German names combined with negative adjectives, and (4) German names combined with positive adjectives. Names and adjectives appeared in capital letters in the center of the screen for 2,000 ms or until response. Participants were instructed to press space when go-items (as defined by the response rule in that block) appeared on the screen and to refrain from responding in all other trials. Each block consisted of 8 practice trials and 120 experimental trials, i.e., there were 480 experimental trials in the GNAT. The feedback was provided during practice but not during experimental trials. Stimuli were presented in black on a white background; the font Sans Serif (14 pixels) was used. The order of blocks was randomized across participants. The instructions used in the experiment can be found in online Supplementary material (see Data availability statement). Accuracy and reaction times (RTs) were measured in both the SJ task and the GNAT.

After finishing the GNAT, participants were asked to fill in a demographic questionnaire, including questions about their age, gender, native language, and handedness (Edinburg Handedness Inventory, EHI, Oldfield, 1971). The entire testing session lasted around 30 min. The study was designed and conducted following the guidelines laid down in the Declaration of Helsinki. All participants submitted their informed consent at the beginning of the experiment by clicking on the corresponding checkbox. Participants were recruited among students of the University of Potsdam by using the SONA Participant Pool and reimbursed with course credits.^3^

We defined a required sample size. Nosek and Banaji (2001, Experiment 6) found a large effect for ethnic stereotypes by using the GNAT (*d* = 0.93). Brysbaert (2019) recommends collecting 50 participants in a 2×2 repeated-measures design for detecting an effect of a size *d* = 0.6, and 85 participants in a correlational analysis for detecting an effect of the same size. Taking into account possible drop-outs (around 10% based on our previous experience) in both experiments (GNAT and SJ), and given that the number of drop-outs can be even larger in online studies (*cf.* Cipora et al., 2019), we aimed to collect data from at least 110 participants.

## Analysis and results

### Sensibility judgment task

The goal of using the SJ task was to test the prediction of the body-specificity hypothesis. According to the body-specificity hypothesis, we expected positive (in-group) stereotypes to be associated with faster right-hand responses and negative (out-group) stereotypes to be associated with faster left-hand responses.

In total, 126 university students took part in the online experiment. Data preparation and analyses were done using Microsoft^®^ Excel^®^ for Microsoft 365, R (R Core Team, 2020), and TIBCO Statistica^™^. We excluded from the main sample non-native speakers of German, participants with neurological or psychiatric diseases, those who reported that they had not performed the tasks seriously, those who did not fill out the questionnaires at the end of the session, and participants with incomplete data in the SJ task (*N* = 21 in all these categories). Data of non-right-handed participants (EHI score < −40; *N* = 13) were also removed. Ninety-two participants remained in the sample (72 female, 18 male, 2 non-binary; *Mean* age = 25 years, *SD* = 9; *Mean* EHI score = 92, *SD* = 13). Two participants indicated their ages as 4 and 7 months. Since this obviously was a mistake, we did not consider these two data points when calculating descriptive statistics for age.

**Figure 1:**
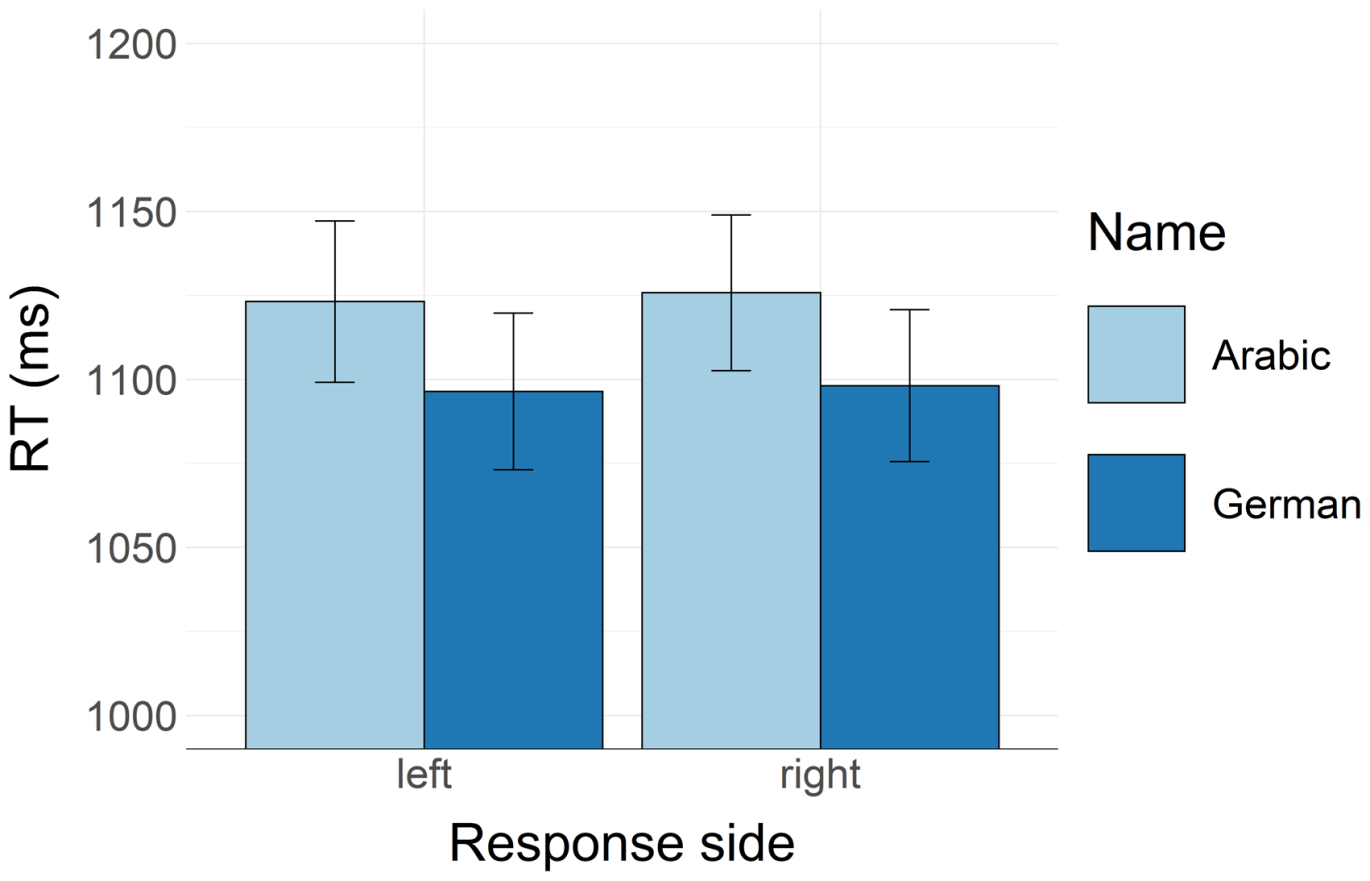
Reaction times in the SJ task aggregated by name and response side. Bars represent mean RT values. Whiskers represent standard errors.

We removed three participants with a mean accuracy of <85% in the SJ task. Only sensible sentences were analyzed (10,680 trials or 100%). Incorrect responses and time-outs (trials with no response) were removed (683 trials or 6% of the data). No error analysis was conducted in the SJ task. Response anticipations (RTs shorter than 400 ms) and procrastinations (RTs longer than 2,500 ms) were removed (97 trials or 1%). The data were aggregated for each participant by condition and submitted to a two-way repeated measures ANOVA with response side (left/right) x name (German/Arabic) as within-factors. We found a significant main effect of name, with faster RTs to sentences with German names [*F*(1, 88) = 20.170, *p* < 0.001, *ηp*^2^ = 0.186]. There was no significant effect of response side [*F*(1, 88) = 0.033, *p* = 0.856, *ηp*^2^ < 0.001]. Contrary to expectations, there was no reliable interaction between response side and name [*F*(1, 88) = 0.005, *p* = 0.946, *ηp*^2^ < 0.001]. Figure 1 illustrates RTs in all four experimental conditions.

Thus, the main analysis did not find a significant interaction between response side and name. To confirm the absence of this critical interaction, we conducted a Bayesian repeated measures ANOVA using the JASP software 0.16.3 (Love et al., 2019). We used a Cauchy prior width of 0.707. The results showed strong evidence in favor of the null effect of the interaction between response side and name (BF_01_ = 28.097). See Table B1 in Appendix B (see Supplementary material) for detailed statistics. We also conducted an additional analysis removing outliers lying +/− 3 standard deviations away from the individual mean by participant by condition. It resulted in the removal of 97 trials (less than 1% of the data) and did not affect the output of the main analysis.

To summarize, the SJ task failed to demonstrate the association between positive (in-group) stereotypes and faster right-hand responses and negative (out-group) stereotypes and faster left-hand responses predicted by the body-specificity hypothesis.

### GNAT 

We used the GNAT as a well-established control instrument for assessing stereotypes in our participants.

One hundred twenty-three students participated in the GNAT study immediately after performing the SJ task; note that three participants quit the experiment after the SJ task. As before, we excluded from the main sample data of non-native speakers of German, participants with neurological or psychiatric diseases, those who reported that they had not performed the tasks seriously, those who did not fill out the questionnaires at the end of the session, and participants with incomplete data in the GNAT (*N* = 18 in all these categories). Data of non-right-handed participants (EHI score < −40; *N* = 13) were also removed. Ninety-two participants remained in the sample (72 female, 18 male, 2 non-binary, *Mean* age = 22 years, *SD* = 9, *Mean* EHI score = 92, *SD* = 13).

The mean accuracy of the participants was 88%. We excluded data from eight participants whose mean accuracy was below 75%.

#### Analysis of accuracy: The sensitivity index (d-prime)

**Figure 2:**
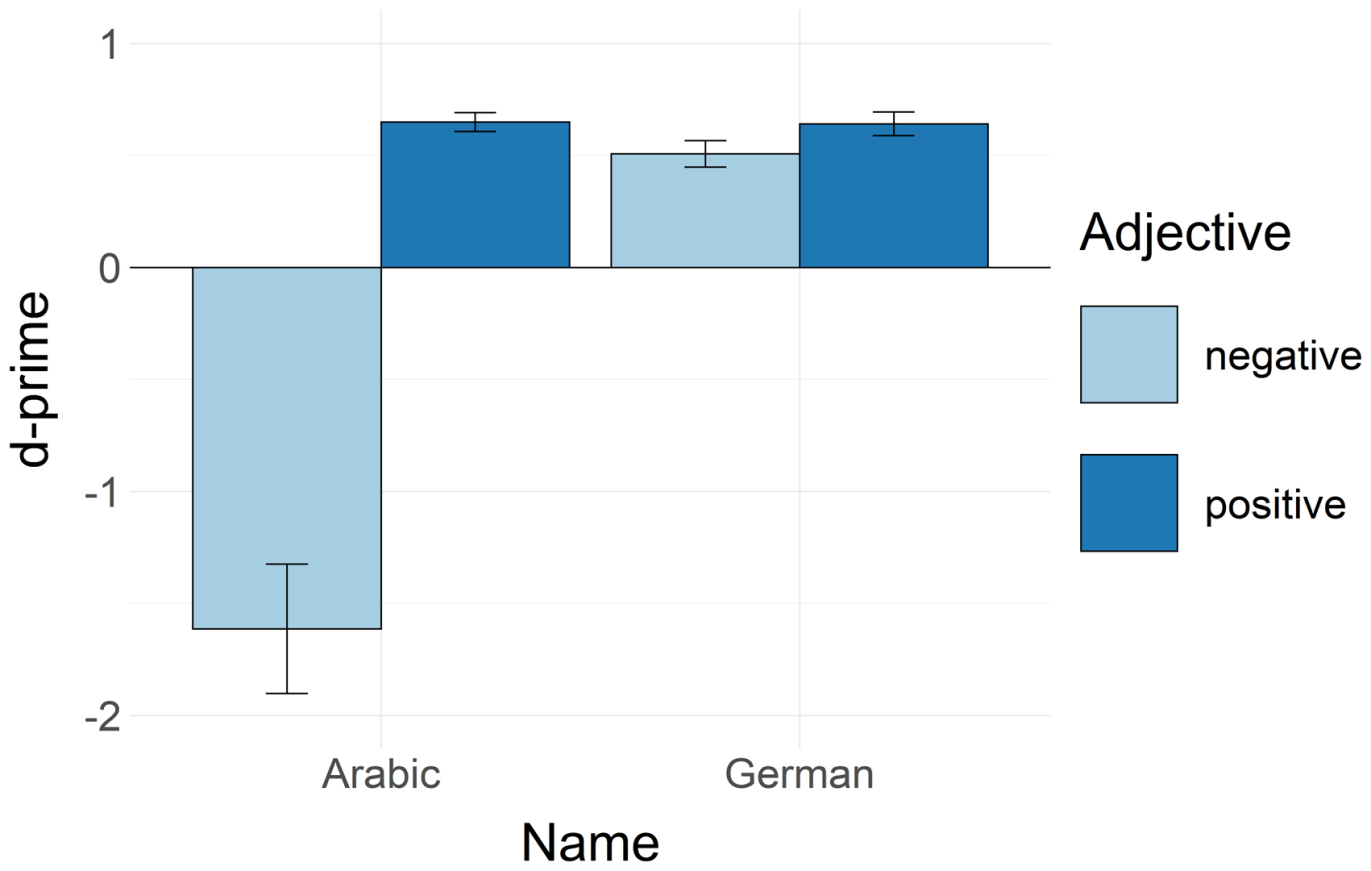
D-primes in the GNAT aggregated by name and adjective. Bars represent mean values. Whiskers represent standard errors. D-prime values of 0 or below indicate that participants were unable to discriminate signal from noise or did not perform the task as instructed.

We calculated the sensitivity index (d-prime) following the method by Nosek and Banaji (2001): (1) we calculated the proportion of hits (correct go-responses) and false alarms (incorrect go-responses) for each participant for each condition; (2) these proportions were z-transformed; (3) the difference (d-prime, or d’) between the z-score values for hits and false alarms was calculated for each participant for each condition. As suggested by Banaji and Greenwald (1995), we applied a formula (0.35/number of trials) for empty cells. We used the function identify_outliers from the rstatix package^4^ in R to identify outliers. Four participants had extreme outliers (defined as data points outside of three inter-quartile ranges from the first and the third quartiles) in at least one of the conditions. The data of these participants were excluded from further analysis. The data of the remaining 80 participants were submitted to a repeated measures ANOVA with name (Arabic/German) and adjective (positive/negative) as within-factors. There was a significant main effect of name [*F*(1, 79) = 49.034, *p* < 0.001, *ηp*^2^ = 0.383] with higher sensitivity to German than to Arabic names. There was a significant main effect of adjective [*F*(1, 79) = 68.352, *p* < 0.001, *ηp*^2^ = 0.464] with higher sensitivity to positive as compared to negative adjectives. We also found a significant interaction between name and adjective [*F*(1, 79) = 59.066, *p* < 0.001, *ηp*^2^ = 0.428]. Further post-hoc analysis was applied to explore this interaction. We found significant differences between responses to Arabic names combined with negative adjectives as compared to all other conditions (all *p*-values <0.001). In contrast, all other conditions were not significantly different from each other. See Figure 2 for descriptive statistics.

We also repeated the analysis without excluding extreme outliers. Neither the qualitative pattern of results nor their significance has changed.

#### Analysis of RTs

**Figure 3:**
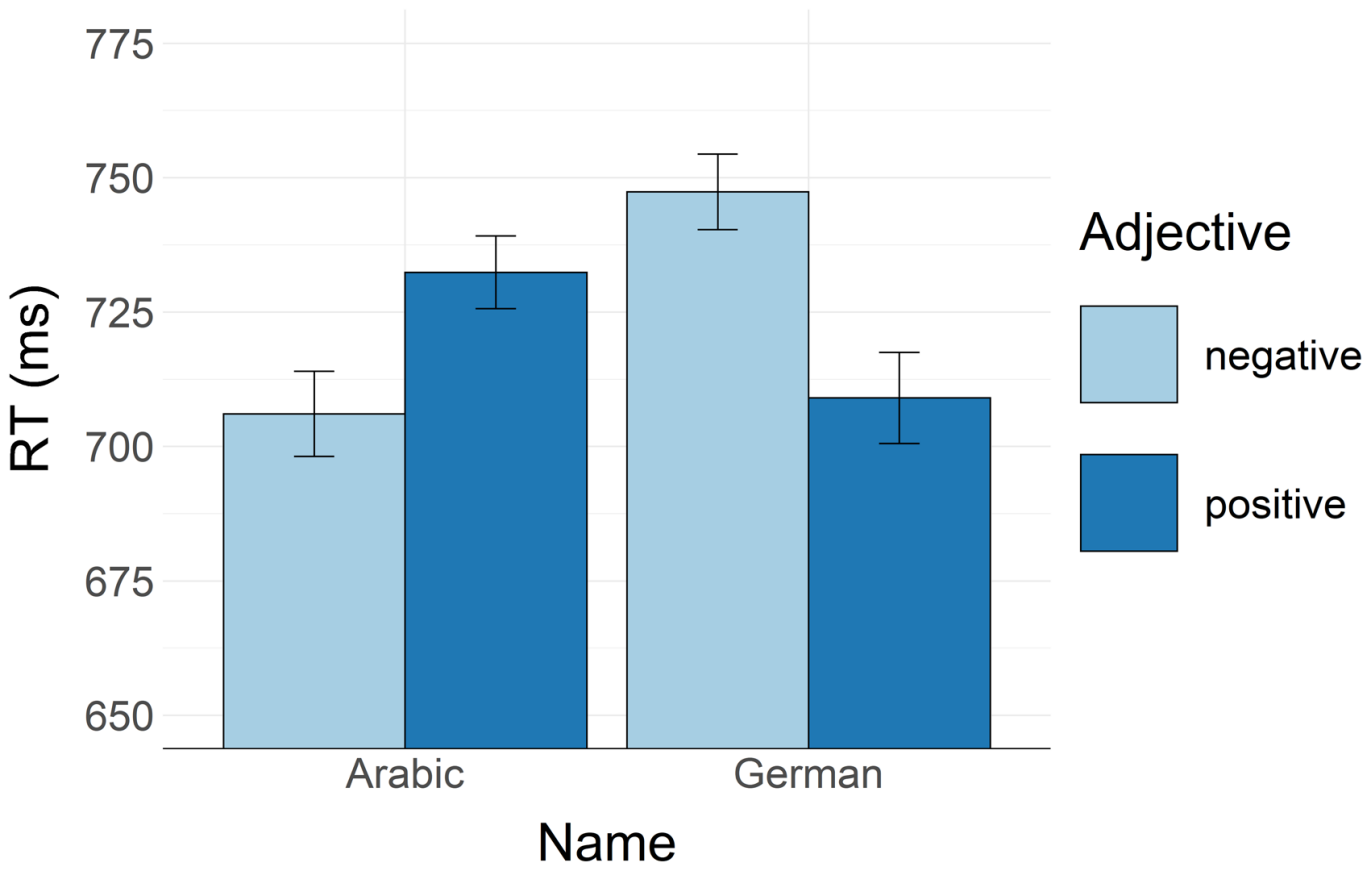
RTs in the GNAT aggregated by name and adjective. Bars represent mean RT values. Whiskers represent standard errors.

All no-go trials were removed. From the remaining 20,160 go-trials (100%), errors were removed (2,146 trials or 11%). Response anticipations (RTs shorter than 400 ms) and procrastinations (RTs longer than 1,200 ms) were removed (1,206 trials or 6%). Thirteen participants had less than five trials in at least one of the conditions, so we excluded them from further analysis. The remaining data of 71 participants were aggregated and submitted to a two-way repeated measures ANOVA with adjective (positive/negative) × name (German/Arabic) as within-participant factors. The analysis yielded a significant main effect of name [*F*(1, 70) = 4.407, *p* = 0.039, *ηp*^2^ = 0.059] with slower RTs to German names compared to Arabic names. There was no significant effect of adjective [*F*(1, 70) = 1.483, *p* = 0.227, *ηp*^2^ = 0.021]. Most importantly, there was a significant interaction between name x adjective [*F*(1, 70) = 49.742, *p* < 0.001, *ηp*^2^ = 0.415]. To explore this interaction, we applied a post-hoc test. As predicted, participants reacted faster to German names combined with positive adjectives as compared to German names combined with negative adjectives (*p* = 0.002). Participants also responded faster to Arabic names combined with negative adjectives as compared to German names combined with negative adjectives (*p <* 0.001). Responses to Arabic names combined with negative adjectives were somewhat faster than responses to Arabic names combined with positive adjectives (*p* = 0.07, i.e., close to significant). All other comparisons were far from significant (*p*-values >0.13). Figure 3 illustrates the descriptive statistics.

Thus, as predicted, in the GNAT, German participants demonstrated faster responses to German names combined with positive as compared to negative adjectives, i.e., positive in-group stereotypes. The participants also responded faster to Arabic names combined with negative adjectives as compared to German names combined with negative adjectives, i.e., in our participants, negative properties were more associated with the out-group than with the in-group. Moreover, there was a tendency to associate the out-group more with negative adjectives than with positive adjectives, i.e., our participants showed marginal evidence of negative out-group stereotypes. Interestingly, in the accuracy analysis, there was a significant difference between responses to Arabic names combined with negative adjectives compared to all other conditions. Namely, our participants demonstrated negative d-prime values when responding to Arabic names combined with negative adjectives. This pattern might reflect additional voluntary control (*cf.* social desirability effects) over reactions in that condition in an attempt to suppress existing negative out-group stereotypes that are evident from the analysis of RTs.

### Comparison across tasks

As stated above, we used the GNAT after the SJ task as a well-established instrument to assess stereotypes in our participants. Thus, we expected our participants to demonstrate similar stereotype effects (i.e., space-valence associations in the SJ task and typical in-vs. out-group valence associations in the GNAT) across both tasks with a significant correlation of those effects at the individual level.

Overall, there were 70 participants included in this correlational analysis after the data exclusion procedures described in previous subsections. For each participant, we calculated congruency scores as the difference between incongruent and congruent conditions. In the formulas below, we use square brackets to denote conditions in the experiments, while parentheses are used in their traditional mathematical sense, i.e., to define the order of calculations.

- SJ_Arabic stereotypes_ = [Arabic name & right response] – [Arabic name & left response] = incongruent condition – congruent condition
- SJ_German stereotypes_ = [German name & left response] – [German name & right response] = incongruent condition – congruent condition
- SJ_congruency_ = ([German name & left response] + [Arabic name & right response]) – ([German name & right response] + [Arabic name & left response]) = (2 incongruent conditions) – (2 congruent conditions)
- GNAT_Arabic stereotypes_ = [Arabic name & positive adjective] – [Arabic name & negative adjective] = incongruent condition – congruent condition
- GNAT_German stereotypes_ = [German name & negative adjective] – [German name & positive adjective] = incongruent condition – congruent condition
- GNAT_congruency_ = ([German name & negative adjective] + [Arabic name & positive adjective]) – ([German name & positive adjective] + [Arabic name & negative adjective]) = (2 incongruent conditions) – (2 congruent conditions)

**Figure 4:**
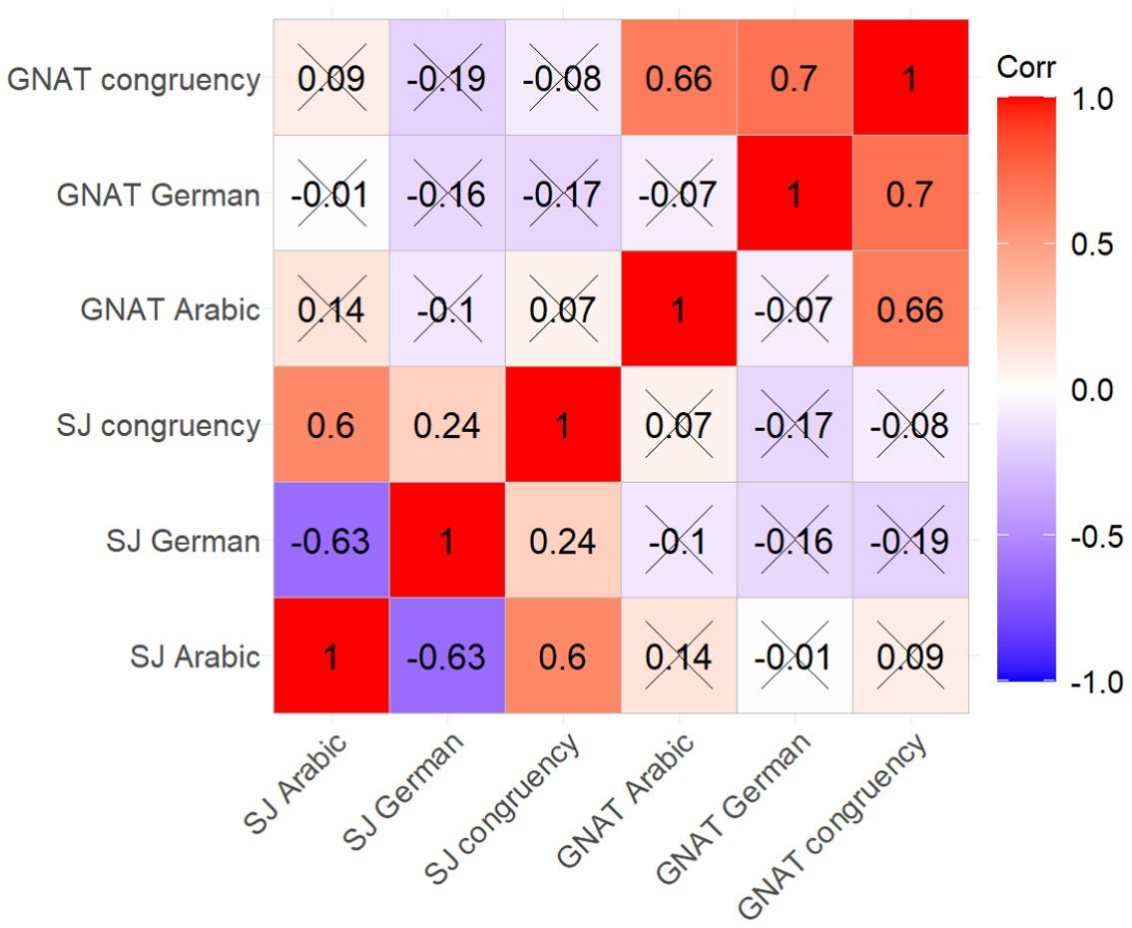
Correlations between congruency variables. Values in each cell represent correlation coefficients. Color coding denotes the direction of the correlation (see legend). Crosses indicate non-significant correlations (*p* > 0.05).

These new variables were submitted to a correlational analysis (see Figure 4).

**Figure 5:**
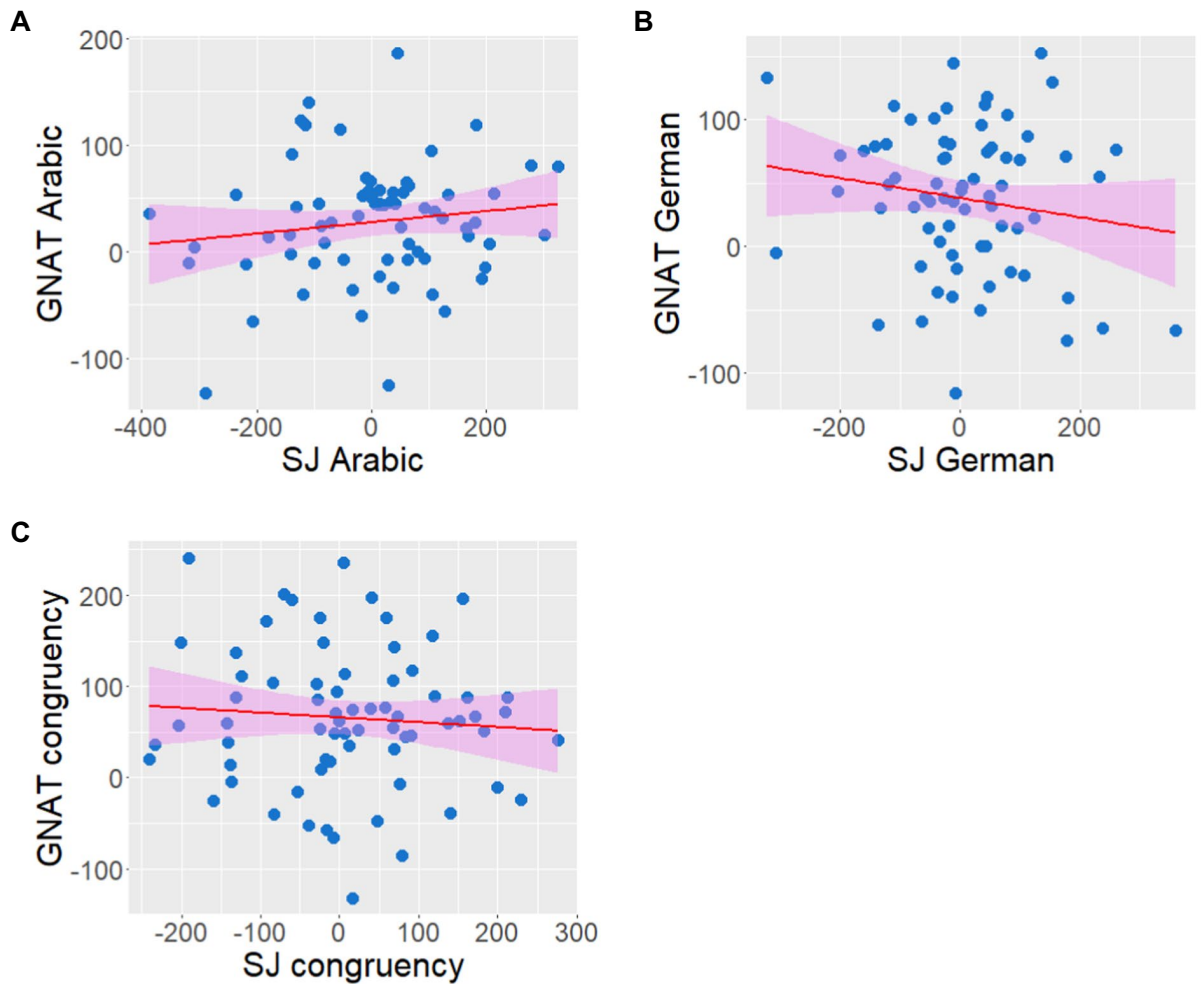
Critical pairwise correlations between SJ and GNAT congruency variables. **A** Correlation between GNAT_Arabic stereotypes_ and SJ_Arabic stereotypes_. **B** Correlation between GNAT_German stereotypes_ and SJ_German stereotypes_. **C** Correlation between GNAT_congruency_ and SJ_congruency_. Red lines represent predictions from linear models. Shaded areas represent the 95% confidence intervals. All variables are in milliseconds. No correlation reached significance. See the main text for details.

As indicated in Figure 4, no significant correlations were found across the three GNAT and three SJ congruency variables. Most important, the correlation between GNAT_Arabic stereotypes_ and SJ_Arabic stereotypes_ was not significant (*r* = 0.140, *p* = 0.247), as well as correlations between GNAT_German stereotypes_ and SJ_German stereotypes_ (*r* = −0.161, *p* = 0.183), and between GNAT_congruency_ and SJ_congruency_ (*r* = −0.079, *p* = 0.516; see Figure 5 for graphic representation of these three correlations).

To summarize, the main analysis did not yield significant correlations across congruency variables from the SJ task and the GNAT. To confirm the absence of these critical correlations, we conducted a series of Bayesian Pearson correlational analyses using the JASP software 0.16.3 with a stretched Beta prior width of 1.0. There was anecdotal to moderate evidence in favor of null effects (BF_01_ ranging from 2.813 to 5.448). See Table B2 in Appendix B (see Supplementary material) for detailed statistics.

Thus, the correlational analysis at the individual level did not reveal any relationship between valenced social stereotypes (as measured by the GNAT) and the space-valence mapping of those stereotypes suggested by the body-specificity hypothesis (as measured by the SJ task). The null findings were corroborated by the results of Bayesian analyses.

### Exploratory analysis for left-handed participants

Since the body-specificity hypothesis predicts a relatively stronger association of positive concepts with the left side in left-handers, one could expect a qualitatively different pattern of results in left-handed participants. To test this prediction, we conducted an exploratory analysis on a previously excluded left-handed subsample. For this analysis, we only included participants whose EHI score was below-40. Nine participants remained in the subsample (1 male, 8 female; *Mean* age = 23 years, *SD* = 4; *Mean* EHI score = −85, *SD* = 16).

We followed the same preprocessing steps described above for right-handers in analyses of the SJ task and GNAT. Below, we provide a brief overview of the results. Interested readers can find corresponding figures in Appendix C (see Supplementary material). All processing scripts are available in the online Supplementary material (see Data availability statement).

In the SJ task, only the main effect of name was significant [*F*(1, 8) = 7.101, *p* = 0.029, *ηp*^2^ = 0.470], with faster RTs to sentences containing German names as compared to Arabic names. The main effect of response side was not significant [*F*(1, 8) = 0.156, *p* = 0.703, *ηp*^2^ = 0.019], as well as the interaction between name and response side [*F*(1, 8) = 0.422, *p* = 0.534, *ηp*^2^ = 0.050]. See Figure C1 in Appendix C (see Supplementary material) for descriptive statistics. This result mirrors the one found for right-handed participants. To prove the absence of the critical interaction between name and response side, we conducted a Bayesian repeated measures ANOVA using the JASP software 0.16.3 with a Cauchy prior width of 0.707. The results yielded moderate evidence in favor of the null effect of the interaction between response side and name (BF_01_ = 3.274). See Table B3 in Appendix B (see Supplementary material) for detailed statistics.

In the analysis of d-primes in the GNAT, we observed a main effect of name close to significance [*F*(1, 8) = 5.150, *p* = 0.053, *ηp*^2^ = 0.392], with higher sensitivity to German than to Arabic names. There was a significant main effect of adjective [*F*(1, 8) = 6.045, *p* = 0.039, *ηp*^2^ = 0.430] with higher sensitivity to positive than to negative adjectives. Finally, there was a significant interaction between name and adjective [*F*(1, 8) = 5.737, *p* = 0.044, *ηp*^2^ = 0.418]. A further post-hoc test demonstrated significant differences between responses to Arabic names combined with negative adjectives compared to all other conditions (all *p*-values <0.011). All other conditions were not significantly different from each other. See Figure C2 in Appendix C (see Supplementary material) for descriptive statistics. Overall, this pattern of results closely resembles the one found in right-handed participants.

In the following analyses, only seven participants were included since two participants had less than five observations in at least one condition. In the analysis of RTs in the GNAT, the main effect of name was not significant [*F*(1, 6) = 1.664, *p* = 0.245, *ηp*^2^ = 0.217]. Also the main effect of adjective [*F*(1, 6) = 1.664, *p* = 0.109, *ηp*^2^ = 0.370] and the interaction between name and adjective [*F*(1, 6) = 3.498, *p* = 0.111, *ηp*^2^ = 0.368] did not reach significance, probably due to the insufficient sample size. Nevertheless, the qualitative pattern of results was very similar to that found in right-handers (see Figure C3 in Appendix C) (see Supplementary material).

New congruency variables were calculated for left-handed participants as described above for right-handers. The body-specificity hypothesis makes opposite predictions for space-valence mapping in left-handers compared to right-handers: positive concepts should be associated stronger with the left side in left-handers, and vice versa. However, we decided to keep the calculation of congruency variables across both subsamples identical to make the results easily comparable. As for right-handers before, no significant correlations were found across the three GNAT and three SJ congruency variables in left-handers. Most important, the correlation between GNAT_Arabic stereotypes_ and SJ_Arabic stereotypes_ variables was not significant (*r* = 0.317, *p* = 0.488), as well as correlations between GNAT_German stereotypes_ and SJ_German stereotypes_ variables (*r* = −0.497, *p* = 0.257), and between GNAT_congruency_ and SJ_congruency_ variables (*r* = 0.208, *p* = 0.655; see Figures C4, C5 in Appendix C) (see Supplementary material).

To confirm the absence of these critical correlations, we conducted a series of Bayesian Pearson correlational analyses using the JASP software 0.16.3 with a stretched Beta prior width of 1.0. There seemed to be anecdotal evidence in favor of null effects (BF_01_ ranging from 1.251 to 2.003). See Table B4 in Appendix B (see Supplementary material) for detailed statistics.

To summarize, the pattern of results in the left-handed subsample was nearly identical to the pattern found in the right-handers. The differences in significance levels or supported Bayesian results can be attributed to the insufficient size of the left-handed sample (*N* = 9). Based on the data from the left-handed subsample, we did not find any evidence that handedness impacts the association of social stereotypes with one or the other body side in the SJ task. There was also no reliable correlation between the effects in the SJ task and the GNAT at the individual level in left-handers. The results of Bayesian analyses also seemed to support the null findings.

## Discussion

The present study examined possible spatial mapping of ethnic in-and out-group stereotypes as predicted by the body-specificity hypothesis (Casasanto, 2011). More specifically, we expected that in our German right-handed participants, positive in-group stereotypes of Germans would be automatically (i.e., without explicit relevance for the task) mapped on the right side, and negative out-group stereotypes of Arabs would be mapped on the left side.

To test this hypothesis, we conducted a sensibility judgment (SJ) task in which participants responded to sentences containing German or Arabic names (e.g., *Charlotte eats a cake.* or *Ibrahim steers a vacuum.*) with a right or left button press. The task was to make a sensibility judgment, i.e., to determine whether a sentence is sensible or not. The SJ task was followed by a Go/No-go Association Task (GNAT, Nosek and Banaji, 2001) in which participants responded to German vs. Arabic names combined with positive vs. negative adjectives.

As predicted, in the GNAT, participants reacted faster to German names combined with positive adjectives and to Arabic names combined with negative adjectives, i.e., our participants demonstrated positive in-group stereotypes and negative out-group stereotypes. However, in the SJ task, we failed to find the predicted automatic association of positive stereotypes with the right side and negative stereotypes with the left side of the body. Participants were merely faster when reacting to German names, regardless of the responding hand. This finding can be accounted for by the familiarity effect (Connine et al., 1990) or frequency effect since German names are more frequent and thus are more familiar to our German participants. Interestingly, this result contrasts the result by Dalmaso et al. (2022), who found faster responses to face images of out-group individuals in both White and Asian participants (see also more details about this study below).

Further exploratory analysis on left-handers demonstrated a nearly identical pattern of results, despite the clear prediction of the body-specificity hypothesis that the results should differ across right-and left-handed participants (Casasanto, 2011). The following Bayesian analyses supported the null findings. Thus, our results provide evidence against the automatic mapping of social stereotypes in horizontal space.

Apparently, our participants did not automatically associate German or Arabic names with a specific body side. More crucially, there was no significant correlation between the congruency scores across both the SJ task and GNAT, which means that the stereotypes found in the GNAT did not correspond to any effects in the SJ task.

Why was no automatic (i.e., irrelevant to the task) spatial mapping of ethnic stereotypes demonstrated in our study? One possible argument could be that our participants did not have strong stereotypes in the first place since they were students at an international university close to Berlin and presumably had a rich cross-cultural experience. However, this argument can be easily ruled out by the results of the GNAT. The findings in the GNAT clearly demonstrated that our participants did have typical in-and out-group ethnic stenotypes: Participants’ reaction times were shorter when they responded to Arabic names combined with negative adjectives and to German names combined with positive adjectives. Interestingly, the accuracy analysis and the respective d-prime scores suggested that the participants tended to suppress their stereotypes, probably due to social desirability (see an unusually low d-prime score for Arabic names combined with negative adjectives in Figure 2). Thus, it cannot be ruled out that, in line with previous studies (e.g., Hahn et al., 2014), our participants were aware of their implicit biases and tried to suppress them.

However, there can be alternative explanations for our null findings in the SJ task. First, it could be the sentence structure that we used that influenced our results. In our task, participants’ attention was always drawn to the end of the sentences where a logical error could appear (e.g., *Karl drives a sofa.* or *Meryem speaks water.*). In all our sentences, the last word was crucial for performing the SJ task, whereas the agent was always mentioned at the beginning of the sentence. While shallow processing of agents could be sufficient to perform the task, deeper processing might be necessary to activate social stereotypes. Further studies manipulating the focus of covert attention should examine this possibility, for example, by making the agent in the sentence relevant for the task (e.g., *The mouse writes a book.* vs. *Meryem writes a book.*).

Second, let us consider the successful demonstration of stereotypes in the GNAT. The GNAT has three essential differences as compared to the SJ task: (1) the instruction focuses participants’ covert attention on valence, (2) it also focuses the attention on ethnicity, and (3) these two instructions are given simultaneously. One of the three elements might be crucial for the stereotype effects to appear. First, it is possible that stereotypes can only demonstrate associations with good and bad when an explicit evaluation or choice between two valenced options is required, as was employed in previous studies (Casasanto, 2009; Casasanto and Henetz, 2012). If this is the case, then embodied effects in previous studies might be related not to the mapping of emotional information itself but appear due to interference at the decision-making level. Second, it could be that in the SJ task, German vs. Arabic names activate valence information only if participants’ attention is directed to the ethnic or cultural aspect of the statement, i.e., the ethnic category is made salient (*cf.* Brusa et al., 2021; but see also Dalmaso et al., 2022, for mixed results). It could be achieved by employing a task requiring an explicit judgment about the ethnic category, as was the case in our GNAT. Finally, it is possible that both ethnicity and valence should be in attentional focus *simultaneously* to produce a significant effect (see also Lebois et al., 2015, on the idea that words do not have core semantic features activated automatically). If this is true, then no measurement of stereotypes is possible without explicitly drawing participants’ attention to the presumably hidden aim of the experiment. Each of these three possibilities should be examined in future studies.

Our results align with those shown in a study by de la Vega et al. (2012), who tested space-valence associations using linguistic stimuli. They found that space-valence associations only emerge when response keys are lateralized and valence is explicitly evaluated (see also Song et al., 2017), but not when shallow lexical processing is required (as in a lexical decision task, i.e., when words and pseudowords should be distinguished) or when only one response key is used. Note, however, that our SJ task is substantially different from the lexical decision task used by de la Vega et al. (2012): Our SJ task requires deeper semantic processing and presents concepts in context – two factors that are known to facilitate embodied effects (Louwerse and Jeuniaux, 2008; see for review; Meteyard et al., 2012). The missing space-valence association in the study by de la Vega et al. (2012, Experiment 1) could potentially result from the shallow lexical decision task itself and not from other factors. However, our finding corroborates their conclusion that the valence-space associations could emerge only when explicit attention is drawn to the valence of concepts.

A very recent study by Dalmaso et al. (2022) examined spatial associations of in-and out-group stereotypes in Italian and Asian participants. In two experiments, Japanese and Italian students were asked to categorize images of faces presented on the screen as belonging to one of the two ethnic groups, Asian or White, by pressing either a left-side or right-side key. In both groups of participants, evidence of an association between space and ethnic group was found. More specifically, both Japanese and Italian participants responded faster to Asian faces with the right key than with the left key. In contrast, no differences in responses to White faces were found regardless of participant group and response side. Although this finding demonstrates possible space-valence associations in the social domain, it does not prove automatic mapping of in-and out-group ethnic stereotypes onto horizontal space: Despite predictions of the body-specificity hypothesis, Italian participants demonstrated faster responses to Asian faces with the right key than with the left key. Thus, the exact cognitive mechanisms behind this finding remain unclear.

Notably, unlike our study with lexical stimuli, the study by Dalmaso et al. (2022) employed face images as stimuli. Processing of face stimuli might result in a stronger attentional focus on ethnic features. Moreover, Dalmaso and colleagues explicitly asked participants to categorize ethnicity of facial stimuli. As we discussed above, this explicit task might also lead to the activation of space-valence associations.

## Conclusion

Our results suggest that ethnic stereotypes are not automatically mapped in the horizontal space, contrary to the predictions of the body-specificity hypothesis. While we still cannot completely exclude the possibility that a stronger attentional focus on agents (i.e., stereotypes) in our stimuli material would activate space-valence mapping, it is more plausible that one of the following three factors accounts for our results: (1) participants might need to focus on valence explicitly, (2) participants might need to focus on ethnicity explicitly, or (3) both former conditions must be in place for the space-valence mapping to emerge. Investigation of embodied mapping of stereotypes could become fruitful revenue for future educational and political interventions.

## Data availability statement

The datasets presented in this study can be found in online repositories. The names of the repository/repositories and accession number(s) can be found at: https://osf.io/ft2sj.

## Ethics statement

Ethical review and approval were not required for the study on human participants in accordance with the local legislation and institutional requirements. The patients/participants provided their written informed consent to participate in this study.

## Author contributions

KK and AM made the conception and design of the study, created the stimuli, programmed the experiment, conducted the study, and performed the analysis. KK, AM, and KN wrote, discussed, and revised drafts of the manuscripts before approving the final version. All authors contributed to the article and approved the submitted version.

## Funding

The work of KK was supported by the DFG grant FI 1915/5-2 “Motor priming from an embodied cognition perspective.” We acknowledge the support of the Deutsche Forschungsgemeinschaft (DFG), project number 491466077, and Open Access Publishing Fund of the University of Potsdam. The funders had no role in study design, data collection, analysis, publication decision, or manuscript preparation.

## Conflict of interest

The authors declare that the research was conducted in the absence of any commercial or financial relationships that could be construed as a potential conflict of interest.

## Publisher’s note

All claims expressed in this article are solely those of the authors and do not necessarily represent those of their affiliated organizations, or those of the publisher, the editors and the reviewers. Any product that may be evaluated in this article, or claim that may be made by its manufacturer, is not guaranteed or endorsed by the publisher.

## Footnotes

^1^We use here the terminology originally used by the authors.

^2^ www.gorilla.sc

^3^ https://www.uni-potsdam.de/en/sona-kogwis/index

^4^ https://cran.r-project.org/web/packages/rstatix/index.html
