# Supplementary material for: Space-valence mapping of social concepts: Do we arrange negative and positive ethnic stereotypes from left to right?
Source: Front Psychol. 2022 Dec 9;13:1070177. doi: 10.3389/fpsyg.2022.1070177 (PMC9780541; doi:10.3389/fpsyg.2022.1070177)
Supplement: Supplementary file 1 [file Data_Sheet_1.pdf]

Appendix A

Stimuli materials

Table A1

List of Arabic and German names used in the SJ task and the GNAT

Name	Gender	Origin	Name	Gender	Origin
Ahmet	Male	Arabic	Anna	Female	German
Ali	Male	Arabic	Ben	Male	German
Aliye	Female	Arabic/Hebrew	Charlotte	Female	German
Amina	Female	Arabic	Emil	Male	German
Aslı	Female	Turkish	Emilia	Female	German
Aylin	Female	Turkish	Emma	Female	German
Ayşe	Female	Arabic	Felix	Male	German
Cem	Male	Arabic	Finn	Male	German
Cemal	Male	Arabic	Frieda	Female	German
Cengiz	Male	Turkish	Hannah	Female	German
Dilara	Female	Persian	Henri	Male	German
Elif	Female	Arabic	Jan	Male	German
Emre	Male	Turkish	Johanna	Female	German
Enes	Male	Arabic	Jonas	Male	German
Erol	Male	Turkish	Katharina	Female	German
Fatme	Female	Arabic	Klara	Female	German
Furkan	Male	Arabic	Laura	Female	German

Gülcan	Female	Persian		Lena	Female	German
Hasan	Male	Arabic		Leon	Male	German
Ibrahim	Male	Hebrew		Lisa	Female	German
Sinem	Female	Turkish/Persian		Luis	Male	German
Mehmet	Male	Arabic		Lukas	Male	German
Melek	Female	Arabic		Marie	Female	German
Meryem	Female	Arabic		Max	Male	German
Mustafa	Male	Arabic		Mia	Female	German
Samira	Female	Arabic/Persian		Nico	Male	German
Yağmur	Female (also male)	Turkish		Noah	Male	German
Yasin	Male	Arabic		Oskar	Male	German
Yusuf	Male	Arabic		Paul	Male	German
Zeynep	Female	Arabic		Sophie	Female	German

Table A2

List of sensible sentences used in the SJ task

**Original sentence (sensible)**	**English Translation**
geht zur Tür.	goes to the door.
bläst den Luftballon auf.	inflates a balloon.
fährt ein Motorrad.	rides a motorcycle.
lädt das Handy auf.	charges the mobile phone.
eröffnet ein Restaurant.	opens up a restaurant.

schläft im Bett.	sleeps in bed.
berät einen Politiker.	advises a politician.
hört einen Ton.	hears a sound.
baut das Haus.	builds the house.
erzählt einen Witz.	tells a joke.
wirft einen Ball.	throws a ball.
reitet auf einem Pferd.	rides a horse.
arbeitet in der Bäckerei.	works in the bakery.
gießt die Blumen.	waters the flowers.
verwirft einen Plan.	discards a plan.
besucht die Oma.	visits the grandma.
verhindert die Katastrophe.	prevents a catastrophe.
lackiert das Auto.	painted the car.
fängt einen Fisch.	catches a fish.
fliegt ein Flugzeug.	flies an airplane.
sät einen Baum.	sows a tree.
zitiert den Aufsatz.	cites an essay.
heftet die Blätter ab.	files away the pages.
trägt einen Hut.	wears a hat.
sticht sich ein Tattoo.	pricks a tattoo [gets a tattoo done].
kocht die Nudeln.	cooks the noodles.
rechnet die Matheaufgabe.	calculates the math exercise.

schreibt einen Brief.	writes a letter.
trinkt einen Tee.	drinks a tea.
schließt die Augen.	closes their/the eyes.

Table A3

List of non-sensible sentences used in the SJ task

**Original sentence (non-sensible)**	**English Translation**
geht zur Tabelle.	goes to the chart.
bläst den Kugelschreiber auf.	inflates a ballpoint pen.
fährt ein Gewitter.	rides a thunderstorm.
lädt das Haus auf.	(re)charges the house.
eröffnet ein Dreirad.	opens up a tricycle.
schläft im Mikroskop.	sleeps in the microscope.
berät einen Apfel.	advises an apple.
hört einen Schwamm.	hears a sponge.
baut das Aufgeben.	builds the surrender.
erzählt einen Bagger.	tells an excavator.
wirft einen Planeten.	throws a planet.
reitet auf einem Lichtstrahl.	rides a beam of light.
arbeitet in der Meinung.	works in the opinion.
gießt die Schwester.	waters the sister.
verwirft einen Tisch.	discards a table.
besucht die Freizeit.	visits the free time.

verhindert die Ampel.	prevents the traffic light.
lackiert den Steuerberater.	painted the tax consultant.
fängt einen Strand.	catches a beach.
fliegt ein Fahrrad.	flies a bike.
sät einen Bierkrug.	sows a beer mug.
zitiert den Fensterrahmen.	cites the window frame.
heftet die Socken ab.	files away the socks.
trägt einen Flug.	wears a flight.
sticht sich ein Konzert.	pricks a concert.
kocht die Lampen.	cooks the lamps.
rechnet die Gießkanne.	calculates the watering can.
schreibt einen Topf.	writes a pot.
trinkt einen Handschuh.	drinks a glove.
schließt die Sonne.	closes the sun.

Table A4

List of positive and negative adjectives used in the GNAT (from Bluemke & Friese, 2006)

German word	English translation	Valence
dynamisch	dynamic	positive
eigenständig	self-contained	positive
erfolgreich	successful	positive
flexibel	flexible	positive
individuell	individualistic	positive

begabt	talented	positive
ehrlich	honest	positive
freundlich	friendly	positive
friedlich	peaceful	positive
hoffnungsvoll	hopeful	positive
lebensfroh	full of the joys of live	positive
bescheiden	modest	positive
einfach	simple	positive
familiär	family-loving	positive
gastfreundlich	hospitable	positive
geduldig	patient	positive
gemeinschaftlich	sociable	positive
optimistisch	optimistic	positive
selbstständig	self-dependent	positive
selbstbewusst	self-confident	positive
leidenschaftlich	passionate	positive
musikalisch	musical	positive
nett	nice	positive
tierlieb	animal-loving	positive
genügsam	frugal	positive
hilfsbereit	helpful	positive
natürlich	natural	positive

multikulturell	multicultural	positive
weltoffen	cosmopolitan	positive
idealistisch	idealistic	positive
arrogant	arrogant	negative
geldgeil	greedy	negative
hektisch	hectic	negative
hochmütig	haughty	negative
karrieregeil	strong need for career	negative
machohaft	macho	negative
selbstgefällig	self-satisfied	negative
überheblich	presumptuous	negative
unkollegial	uncooperative	negative
unpersönlich	impersonal	negative
feindselig	hostile	negative
gefühlslos	emotionless	negative
geizig	stingy	negative
krank	ill	negative
nörgelig	peevish	negative
taktlos	tactless	negative
unbeherrscht	uncontrolled	negative
untreu	unfaithful	negative
verantwortungslos	irresponsible	negative

verlogen	lying	negative
abhängig	dependent	negative
arbeitslos	unemployed	negative
hoffnungslos	hopeless	negative
neidisch	envious	negative
pessimistisch	pessimistic	negative
ausländerfeindlich	xenophobic	negative
trist	sad	negative
rechtsradikal	right wing extremist	negative
unproduktiv	non-productive	negative
unzufrieden	unsatisfied	negative
