# Supplementary material for: Space-valence mapping of social concepts: Do we arrange negative and positive ethnic stereotypes from left to right?
Source: Front Psychol. 2022 Dec 9;13:1070177. doi: 10.3389/fpsyg.2022.1070177 (PMC9780541; doi:10.3389/fpsyg.2022.1070177)
Supplement: Supplementary file 2 [file Data_Sheet_2.pdf]

Appendix B

Bayesian analyses: Result tables

Table B1

Results of the Bayesian Repeated Measures ANOVA (right-handers, SJ task)

**Models**	**P(M)**	**P(M|data)**	**BFM**	**BF01**	**error %**
Name	0.200	0.792	15.215	1.000	
Name + Side	0.200	0.175	0.848	4.527	4.832
Name + Side + Name*Side	0.200	0.028	0.116	28.097	4.874
Null model (incl. subject and random slopes)	0.200	0.004	0.017	190.943	4.340
Side	0.200	9.361e-4	0.004	845.837	4.691

Note. All models include participant and random slopes for all repeated measures factors.

Table B2

Results of the Bayesian Pearson correlations (right-handers)

**Correlation**			**Pearson's r**	**BF_{01}**
$SJ_{\text{Arabic stereotypes}}$	–	$GNAT_{\text{Arabic stereotypes}}$	0.140	3.473
$SJ_{\text{German stereotypes}}$	–	$GNAT_{\text{German stereotypes}}$	-0.161	2.813
$SJ_{\text{congruency}}$	–	$GNAT_{\text{congruency}}$	-0.079	5.448

Table B3

Results of the Bayesian Repeated Measures ANOVA (left-handers, SJ task)

**Models**	**P(M)**	**P(M|data)**	**BFM**	**BF01**	**error %**
Null model (incl. participant and random slopes)	0.200	0.297	1.688	1.000	
Name	0.200	0.287	1.608	1.035	3.767

Models	P(M)	P(M|data)	BFM	BF01	error %
Side	0.200	0.166	0.798	1.783	5.623
Name + Side	0.200	0.159	0.759	1.861	6.437
Name + Side + Name*Side	0.200	0.091	0.399	3.274	7.217

Note. All models include participant and random slopes for all repeated measures factors.

Table B4

Results of Bayesian Pearson correlations (left-handers)

Correlation			Pearson's r	BF_{01}
$SJ_{Arabic\ stereotypes}$	–	$GNAT_{Arabic\ stereotypes}$	0.317	1.772
$SJ_{German\ stereotypes}$	–	$GNAT_{German\ stereotypes}$	-0.497	1.251
$SJ_{congruency}$	–	$GNAT_{congruency}$	0.208	2.003
