# Supplementary material for: Space-valence mapping of social concepts: Do we arrange negative and positive ethnic stereotypes from left to right?
Source: Front Psychol. 2022 Dec 9;13:1070177. doi: 10.3389/fpsyg.2022.1070177 (PMC9780541; doi:10.3389/fpsyg.2022.1070177)
Supplement: Supplementary file 3 [file Data_Sheet_3.pdf]

Appendix C

Descriptive statistics for exploratory analysis of the left-handed subsample

Figure C1

Reaction times in the SJ task aggregated by Name and Response Side (left-handers)

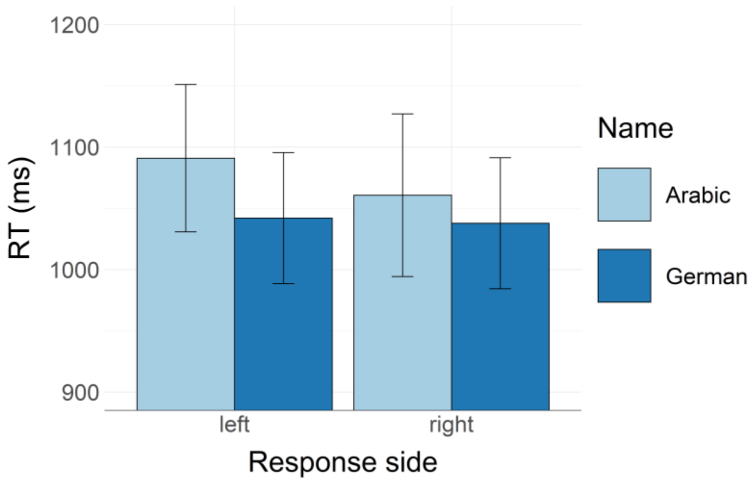

Note. Bars represent mean values. Whiskers represent standard errors.

Figure C2

D-primes in the GNAT aggregated by Name and Adjective (left-handers)

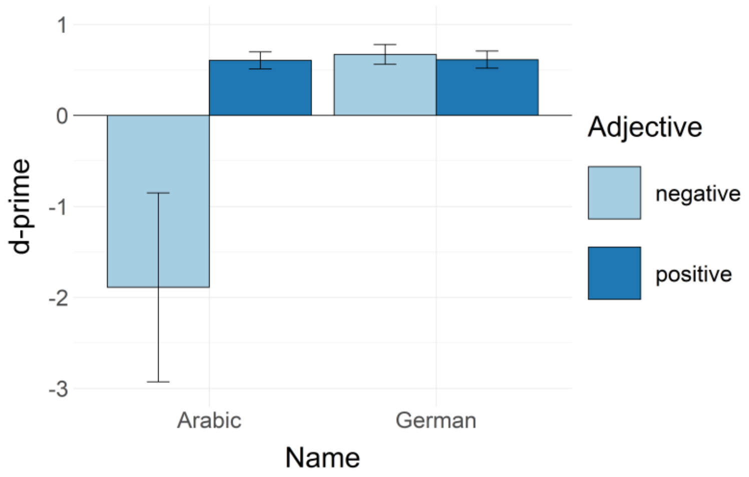

Note. Bars represent mean values. Whiskers represent standard errors.

Figure C3

Reaction times in the GNAT aggregated by Name and Adjective (left-handers)

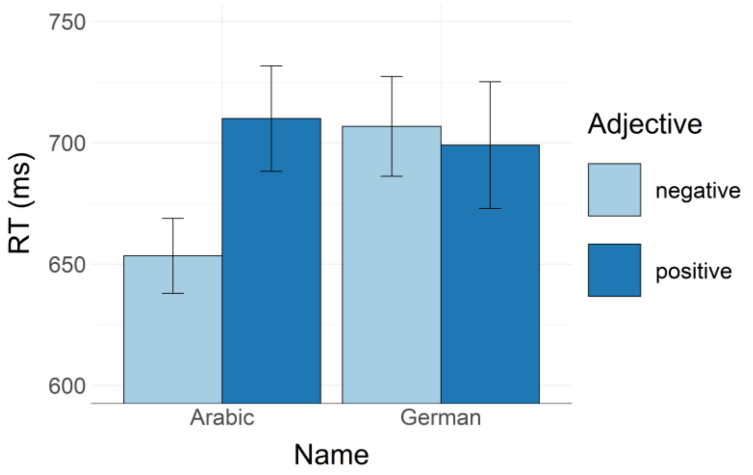

Note. Bars represent mean values. Whiskers represent standard errors.

Figure C4

Correlations between congruency variables (left-handers)

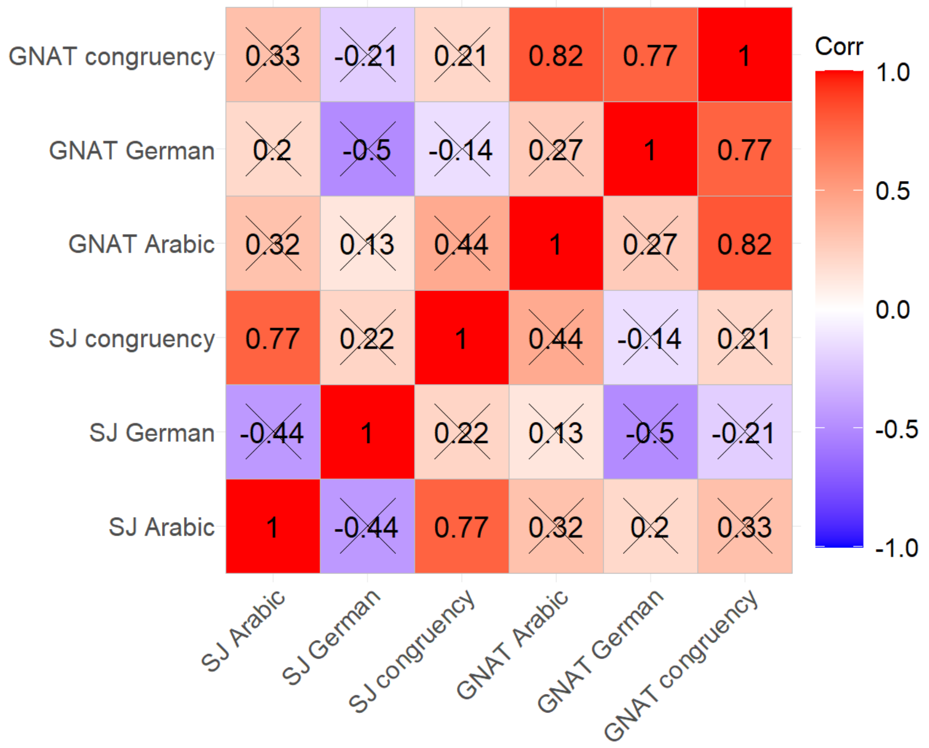

Note. Values in each cell represent correlations coefficients. Color coding denotes the direction of the correlation (see legend). Crosses indicate non-significant correlations (p > .05).

Figure C5

Paired correlations between SJ and GNAT congruency variables (left-handers)

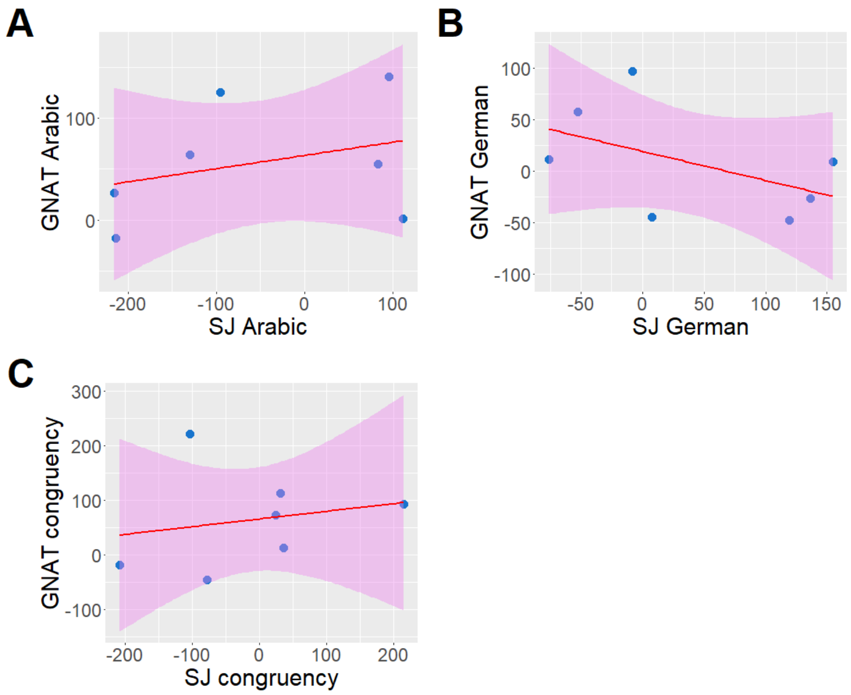

Note. Panel A: correlation between GNAT $_{\text{Arabic stereotypes}}$ and SJ $_{\text{Arabic stereotypes}}$. Panel B: correlation between GNAT $_{\text{German stereotypes}}$ and SJ $_{\text{German stereotypes}}$. Panel C: correlation between GNAT $_{\text{congruency}}$ and SJ $_{\text{congruency}}$. Red lines represent predictions from linear models. Shaded areas represent the 95% confidence intervals. All variabl
